# Molecular Characterization Clinical and Immunotherapeutic Characteristics of m5C Regulator NOP2 Across 33 Cancer Types

**DOI:** 10.3389/fcell.2022.839136

**Published:** 2022-03-16

**Authors:** Taisheng Liu, Jian Zhang, Chunxuan Lin, Guihong Liu, Guofeng Xie, Zili Dai, Peng Yu, Jian Wang, Liyi Guo

**Affiliations:** ^1^ Department of Thoracic Surgery, Affiliated Cancer Hospital and Institute of Guangzhou Medical University, Guangzhou, China; ^2^ Department of Radiation Oncology, Affiliated Cancer Hospital and Institute of Guangzhou Medical University, Guangzhou, China; ^3^ Guangzhou Medical University, Guangzhou, China; ^4^ Department of Pneumology, Guangdong Provincial Hospital of Integrated Traditional Chinese and Western Medicine, Foshan, China; ^5^ Department of Radiation Oncology, DongGuan Tungwah Hospital, Dongguan, China; ^6^ Department of Oncology and Hematology, The Sixth People’s Hospital of Huizhou City, Huiyang Hospital Affiliated to Southern Medical University, Huizhou, China

**Keywords:** NOP2, immunotherapy, pan-cancer, immune response, prognosis

## Abstract

**Background:** Recent studies have identified that RNA 5-methylcytosine (m5C) is a wide-spread epigenetic modification in tumorigenesis. However, the clinical and immunotherapeutic values of m5C regulator NOP2 in 33 cancers remain unclear.

**Methods:** The mRNA expression data and clinical data of 33 cancers were downloaded from The Cancer Genome Atlas (TCGA) database. The immunotherapy data including GSE67501, GSE78220, GSE35640, and IMvigor210 were downloaded from the Gene Expression Omnibus (GEO) database and the website based on the Creative Commons 3.0 license (http://research-pub.Gene.com/imvigor210corebiologies). The expression, survival, clinical parameters, tumor mutation burden (TMB), microsatellite instability (MSI), and tumor microenvironment (TME) were evaluated. Finally, the relationship between NOP2 and immunotherapy response was further explored.

**Results:** NOP2 was significantly upregulated in most cancers, and high NOP2 expression was associated with poor prognosis. TMB, MSI, and NOP2 activities were involved in the dysregulation of NOP2. NOP2 was closely associated with immune cell infiltration, immune modulators, and immunotherapeutic inactivation.

**Conclusion**s**:** We comprehensively explored the clinical and immunotherapeutic values of NOP2 in cancers, providing evidence regarding the function of NOP2 and its role in clinical treatment.

## Introduction

5-Methylcytosine (m5C), one of the RNA methylation modifications, has been identified to be catalyzed by methyltransferases (writers), demethyltransferases (erasers), and readers, which participated in the process of translation and degradation of RNA downstream (Squires et al., 2012; Hussain et al., 2013; Khoddami and Cairns, 2013; Nombela et al., 2021). More than 10,000 sites of m5C modification have been found (Bourgeois et al., 2015), and m5C modification exists in various RNAs, including ribosomal RNA (rRNA), messenger RNA (mRNA), transfer RNA (tRNA), small nuclear RNA, small nucleolar RNA, long non-coding RNA (lncRNA), and microRNA (miRNA) (Sharma et al., 2013; David et al., 2017; García-Vílchez et al., 2019; Winans and Beemon, 2019). Recent studies have reported that m5C was involved in the proliferation and differentiation of tumor cell, tumorigenesis and metastasis, tumor microenvironment (TME), and other biological processes (King and Redman, 2002; David et al., 2017; Cheng et al., 2018a; Pan et al., 2021). However, detailed investigations on the clinical and immunotherapeutic roles of m5C regulators in cancer remain unclear.

Nucleolar protein 2 (NOP2, also NOL1, NOP120, NSUN1, or p120), belongs to the NOP2/SUN (NSUN) RNA methyltransferase family, which also contains six other members (NSUN2-7) (Blanco and Frye, 2014). Emerging research suggests that NOP2 is a multifunctional protein, which plays an important role in RNA modification and maturation (Xue et al., 2020), tumor invasiveness (Kosi et al., 2015), cell cycle progression (Hong et al., 2016), chromatin organization (Cheng et al., 2018b), and HIV latency (Kong et al., 2020). NOP2 has been found to be upregulated in several cancer types, including lung adenocarcinoma (LUAD) (Saijo et al., 2001), leukemia (Cheng et al., 2018a), breast-invasive carcinoma (BRCA) (Freeman et al., 1991), and prostate adenocarcinoma (PRAD) (Bantis et al., 2004), and, therefore, considered to be a predictive cancer marker. Mechanistically, NOP2 binds to the T-cell factor element of cyclin D1 promoter and activates its transcription, thus maintaining cell proliferation capacity (Hong et al., 2016). However, the specific clinical signature and immunotherapeutic characteristics of NOP2 in cancers remain unknown. Thus, clarifying the biological roles of NOP2 in cancers may help identify useful markers for clinical diagnosis and therapeutic treatment.

To understand the potential roles of NOP2 in 33 cancers, we conducted a systematical study on the characteristics of expression, prognosis, and TME of NOP2 from The Cancer Genome Atlas (TCGA) and Gene Expression Omnibus (GEO) databases. We then identified tumor mutation burden (TMB), microsatellite instability (MSI), and NOP2 activity-mediated dysregulation of NOP2. NOP2 was closely associated with immune cell infiltration, immune modulators, and immunotherapeutic inactivation. Overall, our study provides a reliable foundation for detecting new biomarkers for early cancer detection and clinical immunotherapy prediction.

## Materials and Methods

### Dataset Acquisition

The gene expression data and clinicopathological information of 33 human cancers were downloaded from TCGA (https://portal.gdc.cancer.gov/). Three immunotherapeutic cohorts were included in our study: The IMvigor210 cohort (advanced urothelial cancer with atezolizumab intervention) was downloaded from the website based on the Creative Commons 3.0 license (http://research-pub.Gene.com/imvigor210corebiologies) (Mariathasan et al., 2018); the GSE78220 (metastatic melanoma with pembrolizumab treatment), GSE67501 (renal cell carcinoma with nivolumab treatment), and GSE35640 cohorts (metastatic melanoma with MAGE-A3 immunotherapy) were downloaded from the Gene Expression Omnibus database (GEO, https://www.ncbi.nlm.nih.gov/geo/).

### Clinical Correlation Between NOP2 Expression and Pan-Cancer

Gene expression analysis was used to explore the expression difference of NOP2 between normal and tumor tissues using the limma package in R software. The correlation between NOP2 expression and other clinical characters (age, gender, and stage) was further analyzed. Univariate Cox regression analysis was performed using the survival package to investigate the time-dependent prognostic value of NOP2 in cancers. Survival outcomes included overall survival (OS), disease-free survival (DFS), disease-specific survival (DSS), and progression-free survival (PFS). Hazard ratio (HR) > 1 indicated that NOP2 was the promoting factor of death. A statistical two-sided *p* value < 0.05 was considered as having significance.

### Investigation of NOP2 Activity

To detect the NOP2 activity in normal and tumor tissues, single-sample gene set enrichment analysis (ssGSEA) was conducted. The NOP2 activity in normal and tumor tissues was compared. The average expression and activity of NOP2 were calculated and ranked in order in pan-cancer.

### Correlation Analysis Between NOP2 Expression and Immune-Related Factors

To estimate the stromal and immune cells in tumor tissues, the Estimation of STromal and Immune cells in MAlignant Tumor tissues using Expression data (ESTIMATE) algorithm was adopted to calculate the stromal score, immune score, and tumor purity of each patient based on ssGSEA (Yoshihara et al., 2013; Liu et al., 2021a; Liu et al., 2021b; Liu et al., 2021c; Liu et al., 2022a; Liu et al., 2022b). To evaluate the proportion of 22 immune cells, the abundance of immune cell infiltration in the low-NOP2-expressing and high-NOP2-expressing groups was estimated using the cell type identification by estimating the relative subsets of RNA transcripts (CIBERSORT) algorithm (Newman et al., 2015). The correlation between NOP2 expression and TMB and MSI was investigated. Furthermore, the potential relationship between NOP2 expression and immunomodulators (immune inhibitors, immune stimulators, and MHC molecules) was discussed by using the TISIDB website (http://cis.hku.hk/TISIDB/index.php). Then, the four most relevant results were highlighted and displayed in the figure. In the end, Gene Set Enrichment Analysis (GSEA) was performed to infer biological processes between the low-NOP2-expressing and high-NOP2-expressing groups (Subramanian et al., 2005). Adjusted *p* value < 0.05 was considered significant, and the top five highest normalized enrichment scores were considered.

### Immunotherapeutic Response Analysis

As mentioned earlier, three related independent immunotherapeutic cohorts were included and analyzed in this study. Patients who achieved complete remission (CR) or partial response (PR) were classified as responders and compared with non-responders who showed signs of stale disease (SD) or progressive disease (PD). Then, the Wilcoxon test was used to identify the difference of NOP2 expression between the responder group and non-responder group.

## Results

### Clinical Skeleton of NOP2 Expression in 33 Cancers

**FIGURE 1:**
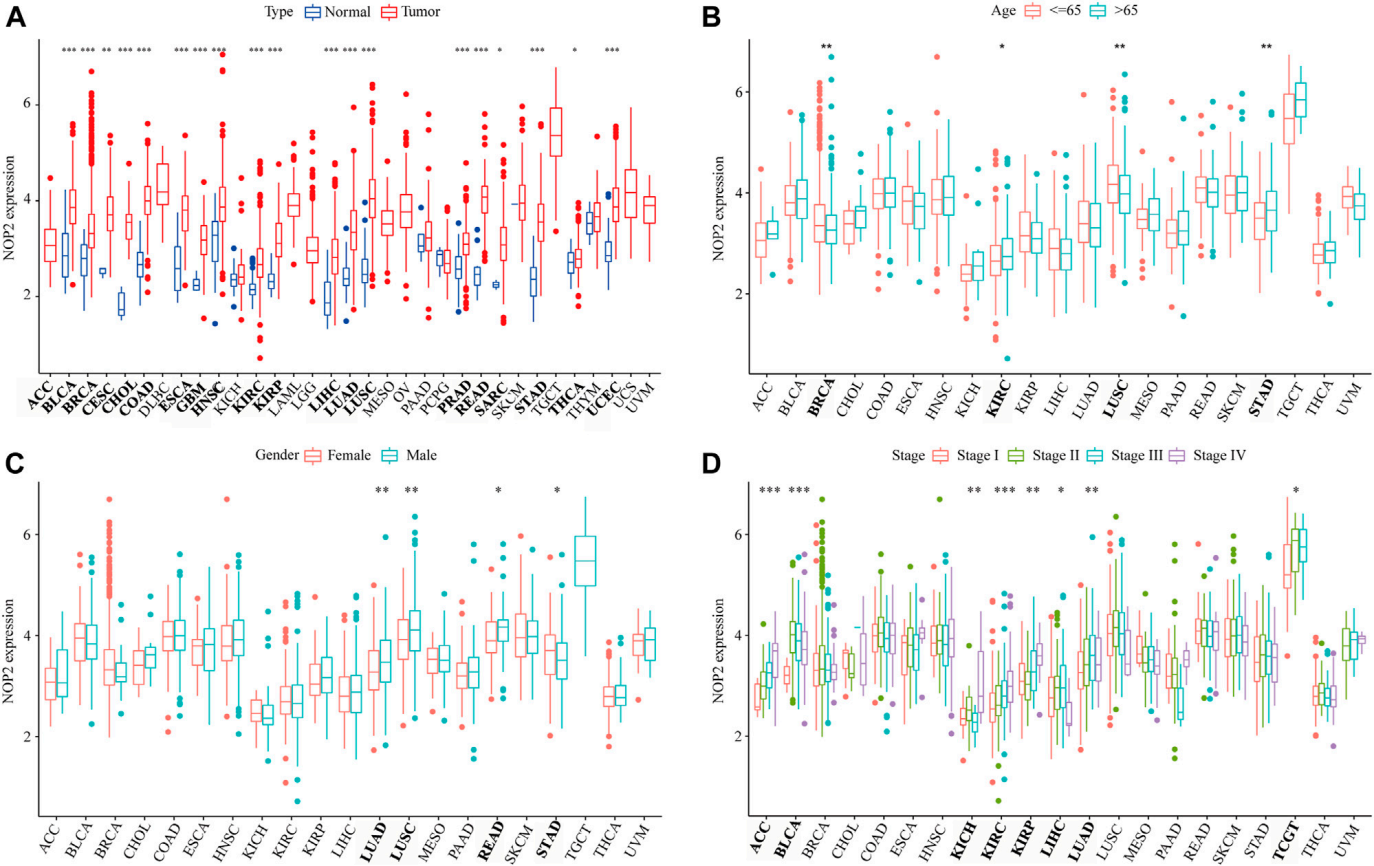
Correlation between clinical characters and NOP2 expression in 33 human cancers. **(A)** Differential expression analysis between the tumor and normal groups of NOP2; **(B)** correlation between age and NOP2; **(C)** correlation between gender and NOP2; and **(D)** correlation between tumor stage and NOP2 (*p < 0.05; **p < 0.01; and ***p < 0.001).

**FIGURE 2:**
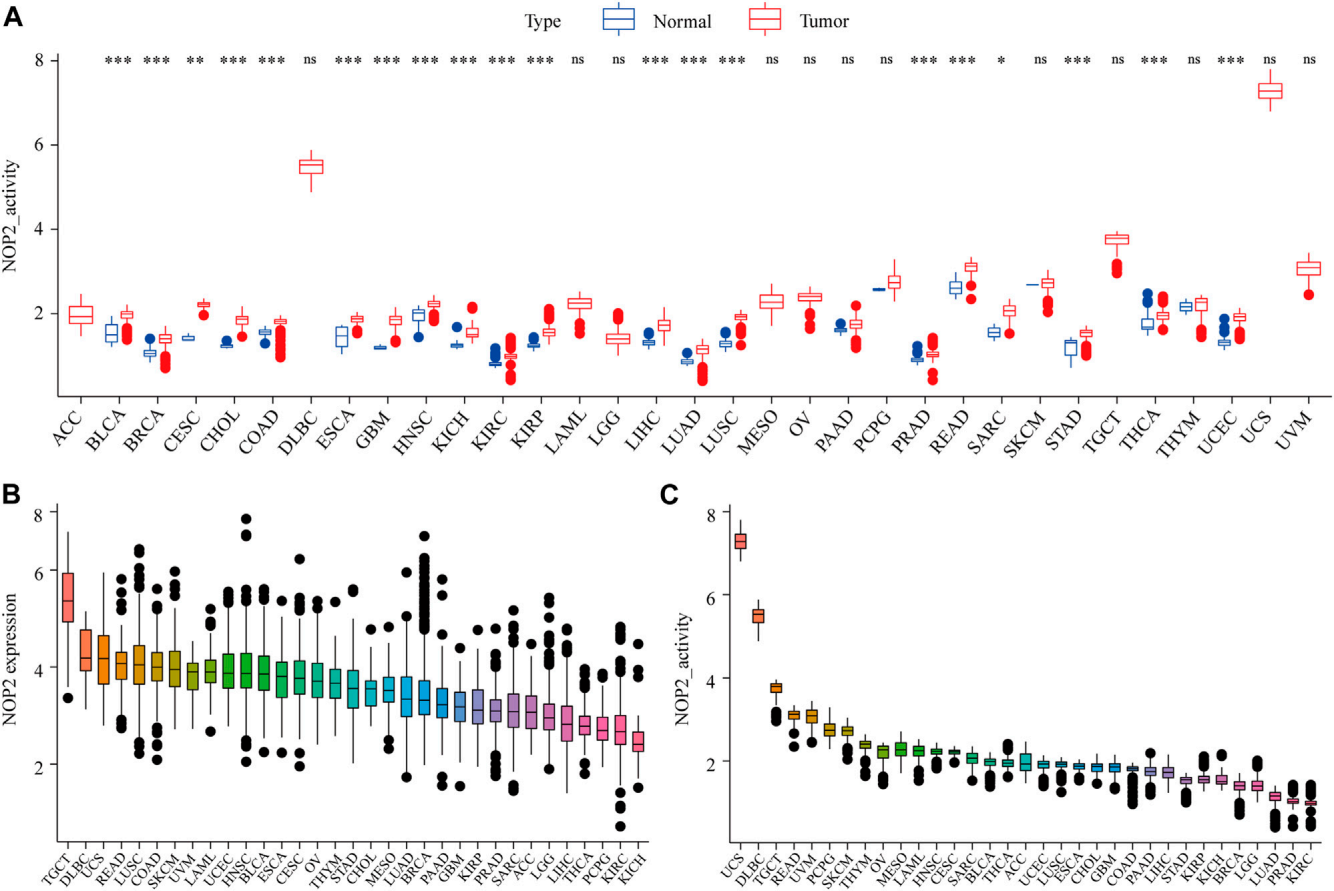
Generation and investigation of NOP2 activity in 33 human cancers. **(A)** Differential activity analysis between the tumor and normal groups of NOP2; **(B)** mean expression of NOP2 in 33 cancers (from high to low); and **(C)** mean activity of NOP2 in 33 cancers (from high to low) (*p < 0.05; **p < 0.01; and ***p < 0.001; ns means no significance).

The detailed analysis process of this article is shown in Supplementary Figure S1. The abbreviations and full names of the 33 cancers in this study are shown in Supplementary Table S1. As shown in Figure 1A, NOP2 was differentially highly expressed in 19 of the 33 cancers (BLCA, BRCA, CESC, CHOL, COAD, ESCA, GBM, HNSC, KIRC, KIRP, LIHC, LUAD, LUSC, PRAD, READ, SARC, STAD, THCA, and UCEC). Subgroup analysis in age showed that NOP2 was differentially highly expressed among younger patients (age ≤ 65) of the BRCA and LUSC groups, whereas it was poorly expressed in the KIRC, LUSC, and STAD samples (age > 65) (Figure 1B). NOP2 expression was significantly correlated with gender such as male in LUAD, LUSC, READ, and STAD (Figure 1C). As illustrated in Figure 1D, there were significant differences in NOP2 expression in different tumor stages of some cancers, such as ACC, BLCA, KICH, KIRC, KIRP, LIHC, and LUAD. As shown in Figure 2A, NOP2 activity was upregulated in most tumor tissues, including BLCA, BRCA, CECS, CHOL, COAD, ESCA, GBM, HNSC, KICH, KIRC, KIRP, LIHC, LUAD, LUSC, PRAD, READ, SARC, STAD, THCA, and UCEC. As shown in Figures 2B,C, the results indicated that NOP2 was highly expressed and active in TGCT, DLBC, and UCS.

### High NOP2 Expression Was Associated With Unfavorable Outcomes in Cancers

**FIGURE 3:**
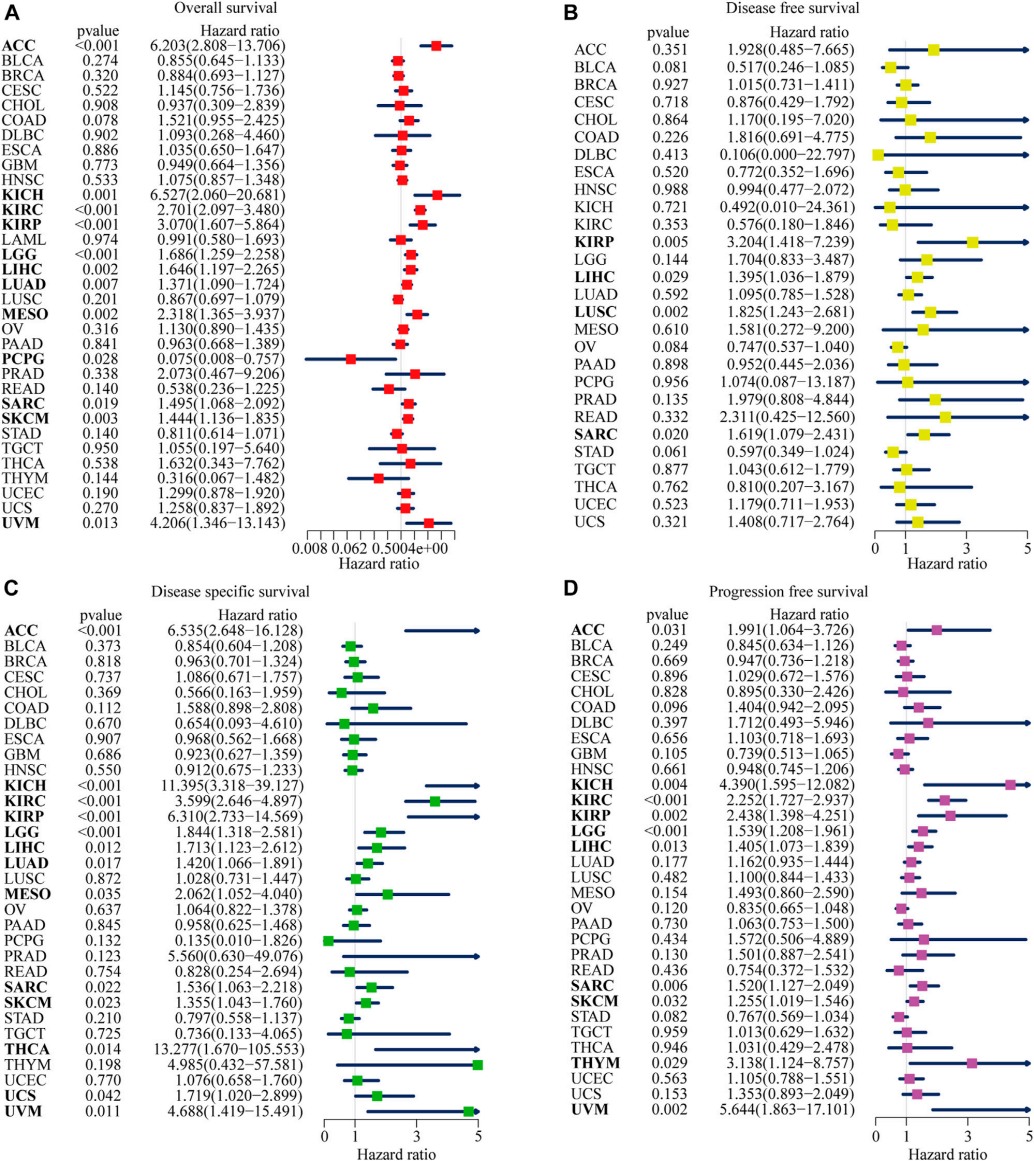
Forest plots of NOP2 expression on the prognosis of various cancers using univariate Cox regression analyses. The bold items mean that NOP2 expression was significantly correlated with prognosis in these types of cancers (*p* < 0.05). **(A)** Effect of NOP2 on OS in 33 types of cancers. **(B)** Effect of NOP2 on DFS in 33 types of cancers. **(C)** Effect of NOP2 on DSS in 33 types of cancers. (D) Effect of NOP2 on PFS in 33 types of cancers.

NOP2 expression was positively correlated with OS in ACC, KICH, KIRC, KIRP, LGG, LIHC, LUAD, MESO, SARC, SKCM, and UVM and negatively correlated in PCPG as shown in Figure 3A and Supplementary Table S2. In terms of DFS, a significant positive association was observed in KIRP, LIHC, LUSC, and SARC as shown in Figure 3B and Supplementary Table S3. Regarding NOP2 in DSS, NOP2 was a risk factor for ACC, KICH, KIRC, KIRP, LGG, LIHC, LUAD, MESO, SARC, SKCM, THCA, UCS, and UVM (Figure 3C and Supplementary Table S4). In addition, as illustrated in Figure 3D and Supplementary Table S5, a positive association was found between NOP2 expression and DFS in ACC, KICH, KIRC, KIRP, LGG, LIHC, SARC, SKCM, THYM, and UVM. Above all, NOP2 expression was not only closely related to clinical parameters but also strongly associated with survival in many types of cancers, including ACC, KICH, KIRC, KIRP, LGG, LIHC, SARC, SKCM, and UVM.

The Kaplan–Meier (KM) curve was used to further visualize the impact of NOP2 on prognosis, and the filter condition was *p* < 0.05. As illustrated in Supplementary Figure S2, higher NOP2 expression indicated worse OS in ACC (*p* < 0.001), KIRC (*p* < 0.001), KIPR (*p* = 0.006), LGG (*p* = 0.001), LUAD (*p* = 0.038), MESO (*p* < 0.001), SARC (*p* = 0.009), SKCM (*p* = 0.006), and UVM (*p* = 0.019). Higher levels of NOP2 expression were also linked with worse DFS in KIRP (*p* = 0.011), LUSC (*p* = 0.004), and OV (*p* = 0.032) (Supplementary Figure S3), worse DSS in ACC (*p* < 0.001), KIRC (*p* < 0.001), KIRP (*p* < 0.001), LGG (*p* = 0.002), MESO (*p* = 0.005), SARC (*p* = 0.012), and UVM (*p* = 0.007) (Supplementary Figure S4), and worse PFS in KIRC (*p* < 0.001), KIRP (*p* = 0.011), LGG (*p* = 0.001), MESO (*p* = 0.030), OV (*p* = 0.002), SKCM (*p* = 0.011), THYM (*p* = 0.030), and UVM (*p* = 0.015) (Supplementary Figure S5).

### Potential Association Between NOP2 Expression and Immune-Related Factors

**FIGURE 4:**
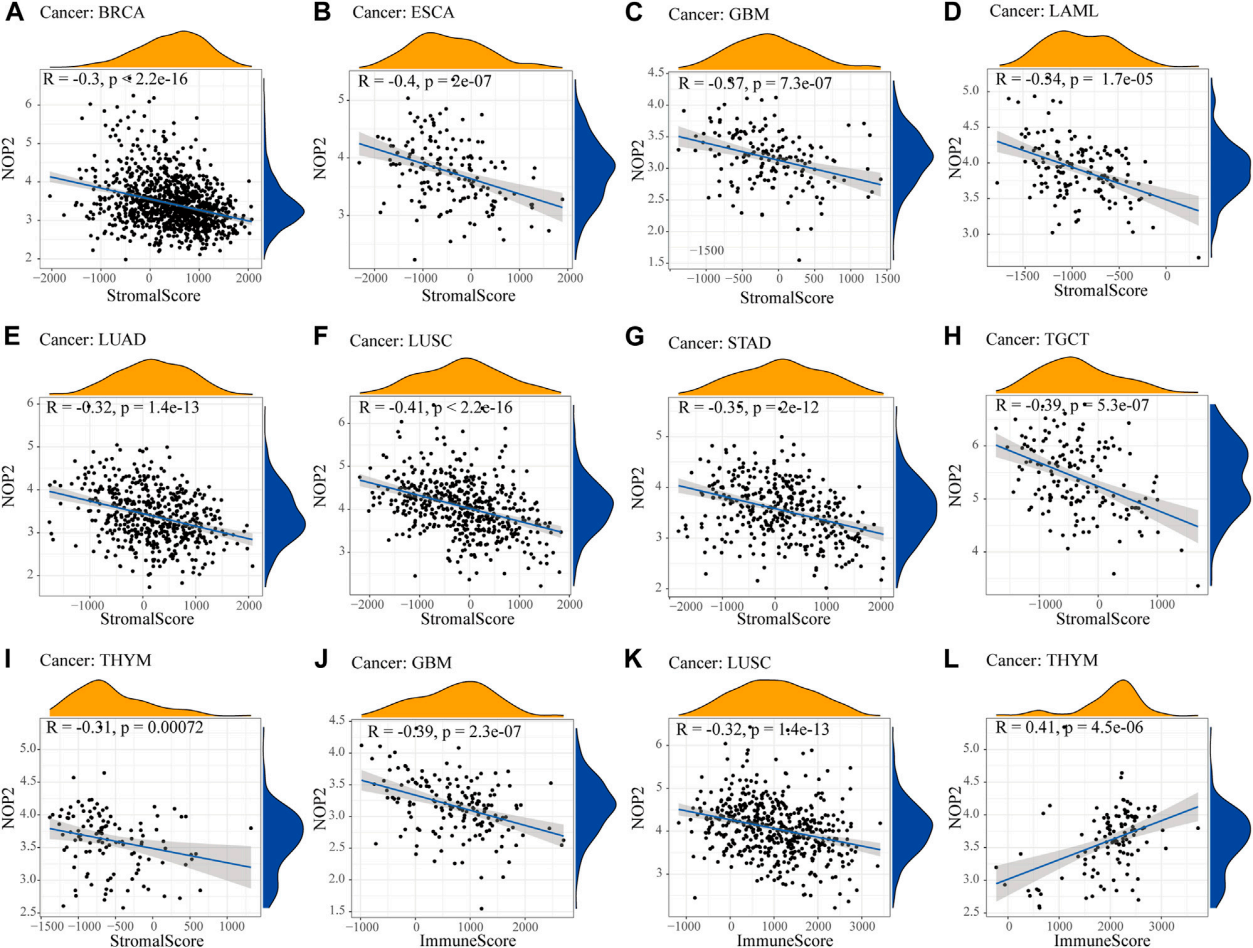
Correlation between NOP2 expression and the ESTIMATE score. The correlation plots are illustrated if R > 0.4 and *p* < 0.05. **(A–I)** Correlation between NOP2 expression and stromal score in BRCA **(A)**, ESCA **(B)**, GBM **(C)**, LAML **(D)**, LUAD **(E)**, LUSC **(F)**, STAD **(G)**, TGCT **(H)**, and THYM **(I)**. **(J–L)** Correlation between NOP2 expression and immune score in GBM **(J)**, LUSC **(K)**, and THYM **(L)**.

**FIGURE 5:**
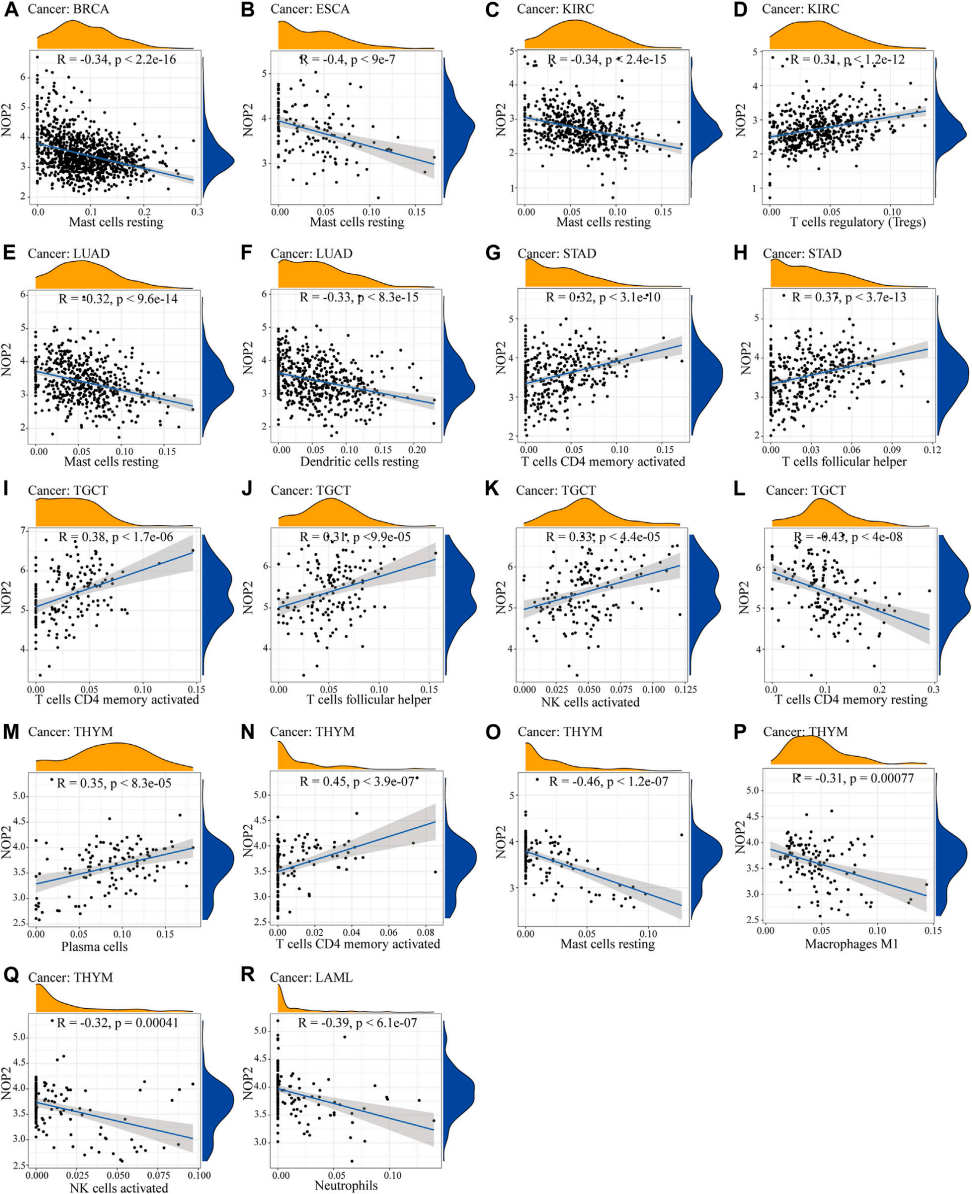
Correlation between NOP2 expression and immune cell infiltration. **(A–C)** Correlation between NOP2 expression and mast cells resting in BRCA **(A)**, ESCA **(B)**, and KIRC**(C)**. **(D)** Regulatory T cells in KIRC. **(E)** Mast cells resting in LUAD. **(F)** Dendritic cells resting in LUAD. **(G–H)** T-cell CD4 memory activated **(G)** and T-cell follicular helper **(H)** in STAD. **(I–Q)** The T-cell CD4 memory activated **(I)**, T-cell follicular helper **(J)**, NK cells activated **(K)**, T-cell memory resting **(L)**, plasma cells **(M)**, T-cell CD4 memory activated **(N)**, mast cells resting **(O)**, macrophage **(P)**, and NK cells activated **(Q)** in THYM. **(R)** Neutrophils in LAML.

**FIGURE 6:**
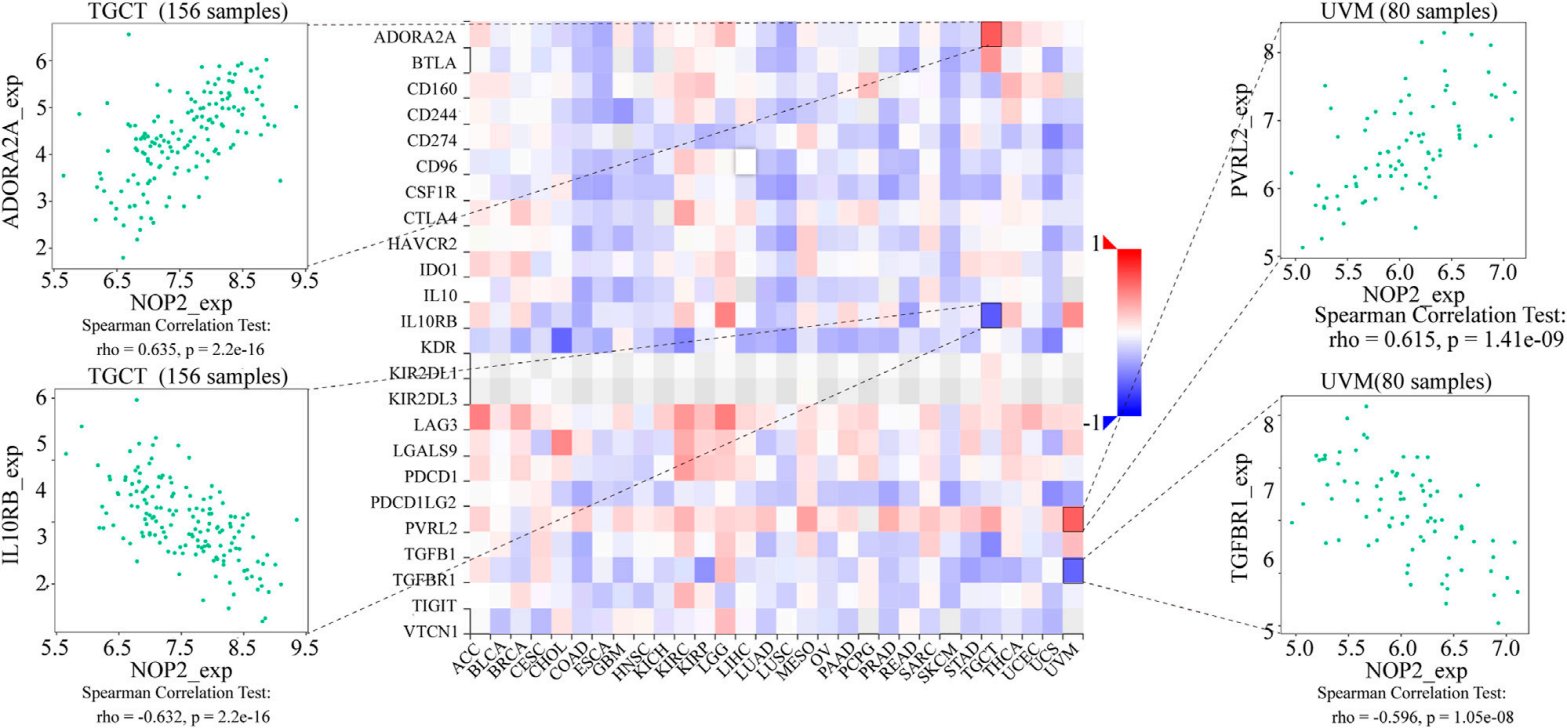
Expression correlation between NOP2 expression and immune inhibitors. Red indicates positive correlation, whereas blue indicates negative correlation. The top four strongest associations are displayed *via* dot plots.

**FIGURE 7:**
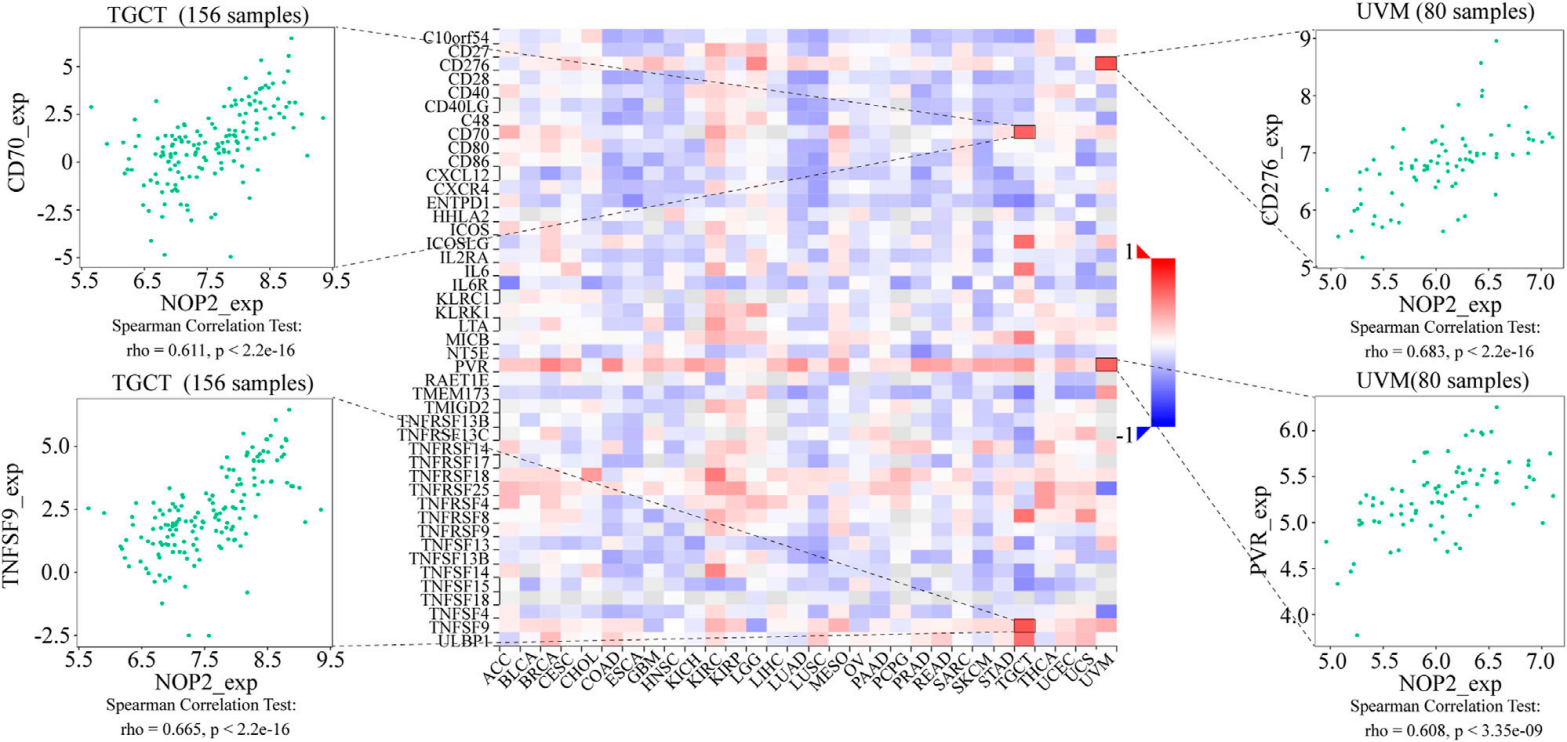
Expression correlation between NOP2 and immune stimulators. Red indicates positive correlation, whereas blue indicates negative correlation. The top four strongest associations are displayed *via* dot plots.

**FIGURE 8:**
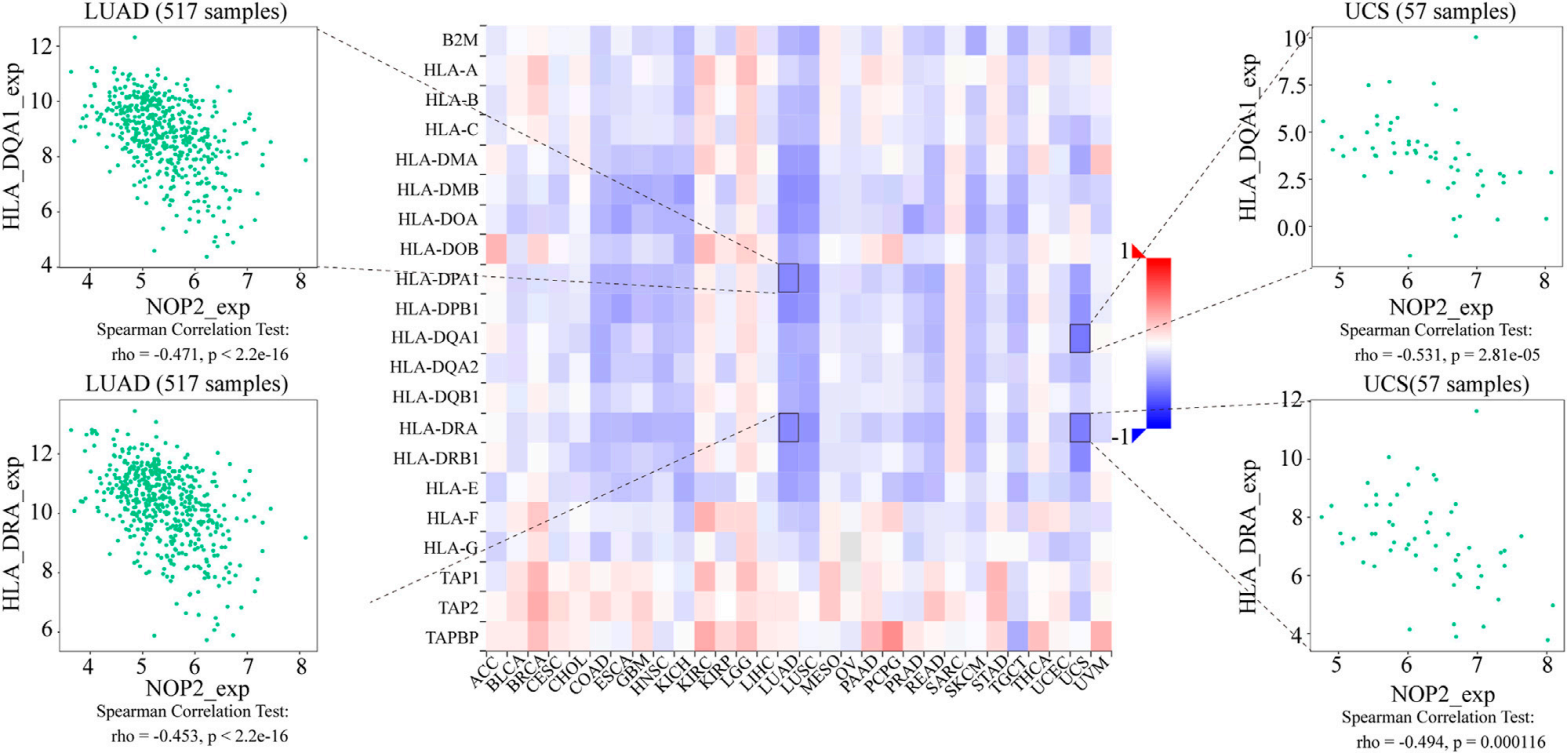
Expression correlation between NOP2 and MHC molecules. Red indicates positive correlation, whereas blue indicates negative correlation. The top four strongest associations are displayed *via* dot plots.

To explore the association between NOP2 expression and immune-related factors, the correlation of NOP2 expression with the stromal and immune scores of the TME using the Spearman rank correlation coefficient in 33 human cancers is summarized in Supplementary Table S6. The stromal score, immune score, and immune cell infiltration are summarized in Figure 4 (*p* < 0.001 and |R| > 0.3). It should be noted that NOP2 expression was negatively correlated with stromal scores in BRCA (R = −0.3, Figure 4A), ESCA (R = −0.4, Figure 4B), GBM (R = −0.57, Figure 4C), LAML (R = −0.34, Figure 4D), LUAD (R = −0.32, Figure 4E), LUSC (R = −0.41, Figure 4F), STAD (R = −0.35, Figure 4G), TGCT (R = −0.39, Figure 4H), and THYM (R = −0.31, Figure 4I). Meanwhile, it was negatively associated to GBM (R = −0.39, Figure 4J) and LUSC (R = −0.32, Figure 4K) and positively associated to THYM (R = 0.41) in immune scores (Figure 4L). Regarding immune cell infiltration, NOP2 expression was negatively associated with mast cells resting in BRCA (R = −0.34, Figure 5A), ESCA (R = −0.4, Figure 5B), and KIRC (R = −0.34, Figure 5C) and was positively associated with regulatory T cells (Tres) in KIRC (R = 0.31, Figure 5D). In LUAD, NOP2 expression was negatively correlated with mast cells resting (R = −0.32, Figure 5E) and dendritic cells resting (R = −0.33, Figure 5F). In STAD, NOP2 expression was positively correlated with T-cell CD4 memory activated (R = 0.32, Figure 5G) and T-cell follicular helper (R = 0.37, Figure 5H). In TCGT, NOP2 expression was positively associated with T-cell CD4 memory activated (R = 0.38, Figure 5I), T-cell follicular helper (R = 0.31, Figure 5J), and NK cells activated (R = 0.33, Figure 5K) and negatively with T-cell CD4 memory resting (R = −0.43, Figure 5L). In THYM, NOP2 expression was positively associated with plasma cells (R = 0.35, Figure 5M) and T-cell CD4 memory activated (R = 0.45, Figure 5N) and negatively with mast cells resting (R = −0.46, Figure 5O), macrophages M1 (R = −0.31, Figure 5P), and NK cells activated (R = −0.32, Figure 5Q). Meanwhile, NOP2 expression was negatively correlated with neutrophils in LAML (R = −0.39, Figure 5R). The relationship between the NOP2 expression and content of 22 immune cells was obtained using the Spearman rank correlation coefficient (Supplementary Table S7). In addition, NOP2 expression was associated with immune modulators. As shown in Figure 6, 24 types of immune inhibitors have been analyzed. NOP2 expression was positively associated with ADORA2A (R = 0.635) and negatively associated with IL10RB (R = −0.632) in TGCT; meanwhile, it was positively associated with PVRL2 (R = 0.615) and negatively associated with TGFBR1 (R = −0.596) in UVM. The NOP2 expression was positively correlated with CD70 (R = 0.611) and TNFSF9 (R = 0.665) in TGCT, as well as CD276 (R = 0.683) and PVR (R = 0.608) in UVM (Figure 7). Furthermore, NOP2 expression was negatively associated with HLA-DQA1 (R = −0.471) and HLA-DRA (R = -0.453) in LUAD; similarly, it was negatively associated with HLA-DQA1 (R = −0.531) and HLA-DRA (R = −0.494) in UVM (Figure 8).

**FIGURE 9:**
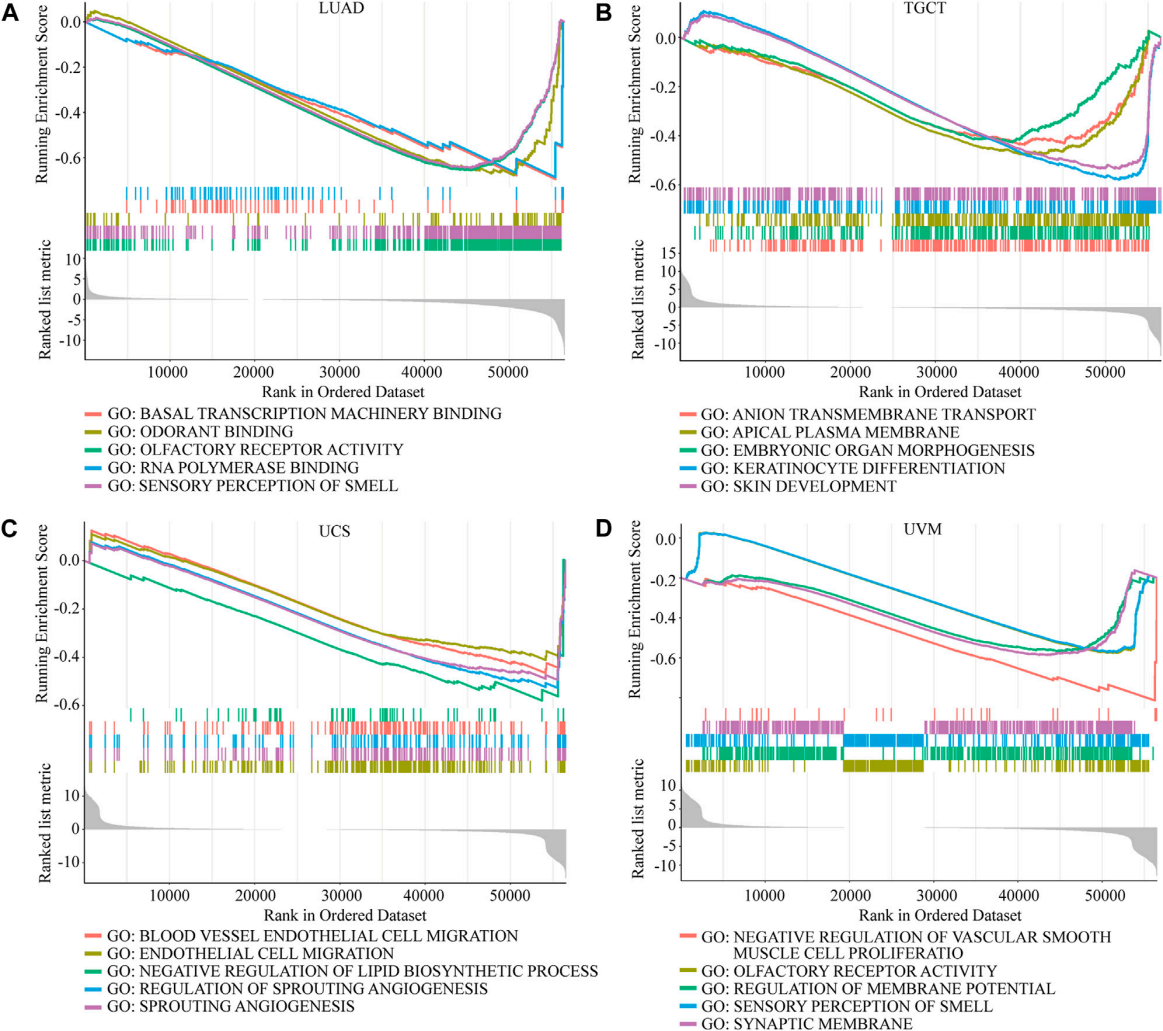
GO enrichment analysis of NOP2 in LUAD, TGCT, UCS, and UVM. Values of p < 0.05 and results higher than five were considered and displayed. **(A)** LUAD. **(B)** TGCT. **(C)** UCS. **(D)** UVM.

**FIGURE 10:**
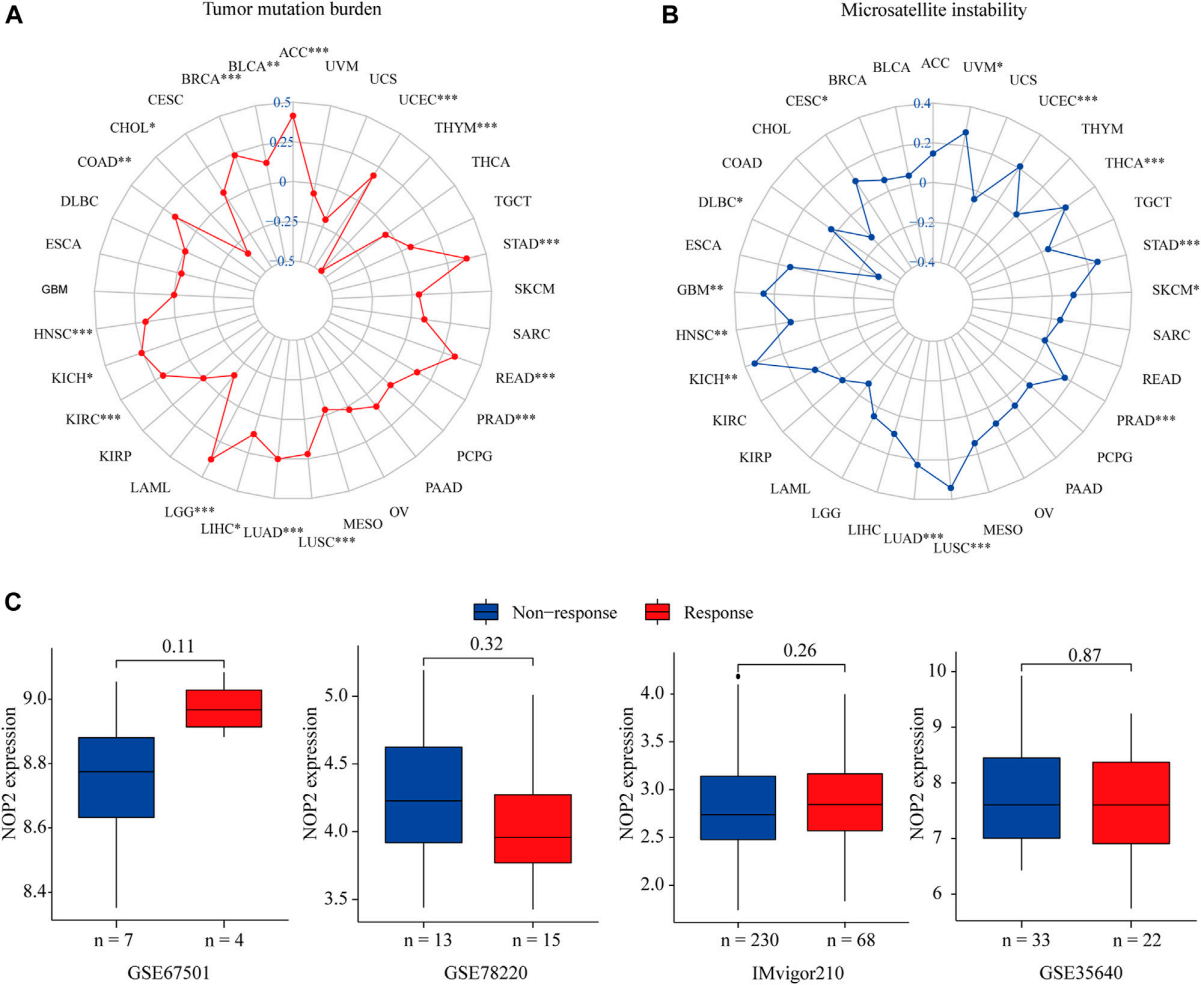
Correlation between NOP2 expression and TMB, MSI, and the immunotherapeutic response in 33 types of cancers. **(A)** Correlations between NOP2 expression and TMB. **(B)** Correlation between NOP2 and MSI. **(C)** Correlations between NOP2 and immunotherapeutic response in GES67501, GSE78220, and IMvigor210.

Considering the strong correlation between NOP2 and LUAD, TCGT, UCS, and UVM, GSEA was performed to explore the roles of the NOP2 signaling pathway in the four types of cancers. Results at *p* < 0.05 were screened out, and the enrichment results higher than five were visualized. Gene Ontology (GO) analysis suggested that NOP2 was mainly concentrated in the olfaction, angiogenesis and regulation, RNA polymerase, and membrane methyltransferase potential and transport in the four types of cancers (Figures 9A–D and Supplementary Tables S8–S11). More results of GO and Kyoto Encyclopedia of Genes and Genomes (KEGG) analysis in other cancers are shown in Supplementary Figures S6, S7, NOP2 was involved in many biological processes including immune-related pathways such as adaptive immune response, regulation of the immune effector process, immune response regulating cell surface receptor signaling, the Jak stat signaling pathway, and toll-like receptor signaling pathway. The correlation between NOP2 and immunotherapy-related biomarkers (TMB and MSI) was further explored. As illustrated in Figure 10A and Supplementary Table S12 **,** NOP2 expression was positively related to the TMB in 15 of 33 cancers, including ACC, BLCA, BRCA, COAD, HNSC, KICH, KIRC, LGG, LIHC, LUAD, LUSC, PRAD, READ, STAD, and UCEC, whereas a negative association was found in CHOL and THYM. NOP2 expression positively correlated significantly with MSI in CESC, GBM, HNSC, KICH, LUAD, LUSC, PRAD, SKCM, STAD, THCA, UCEC, and UVM (*p* < 0.05), while a negative association in DLBC was identified (Figure 10B and Supplementary Table S13). However, as shown in Figure 10C, there was no significant difference in NOP2 expression between the responder and non-responder groups in all three independent cohorts.

## Discussion

NOP2 is a nuclear RNA catalyzing m5C formation. However, the underlying immunotherapeutic roles and molecular mechanisms of NOP2 dysregulation in tumors have not been fully elucidated. In this study, we comprehensively analyzed the expression, prognosis, TME, immune infiltrating cells, immune modulators, and immunotherapeutic response of NOP2 in pan-cancer.

In the current study, we first revealed that NOP2 expression was upregulated in BLCA, BRCA, CESC, CHOL, COAD, ESCA, GBM, HNSC, KIRC, KIRP, LIHC, LUAD, LUSC, PRAD, READ, SARC, STAD, THCA, and UCEC tissues, suggesting that NOP2 might act as an oncogene in pan-cancer. According to the survival analysis of OS, DFS, DSS, and PFS, NOP2 expression has reliable diagnostic value, indicating that NOP2 is a potential biomarker for multiple cancer diagnosis. The correlation between NOP2 expression and clinical parameters revealed that some cancer types had a significant difference in age, gender, and tumor stage. For example, NOP2 expression was statistically different in age ≤ 65 and age > 65 groups in BRCA, KIRC, LUSC, and STAD. NOP2 expression appeared to be higher in male patients, especially in LUAD, LUSC, and READ. A trend was also observed that the later the tumor stage, the higher the expression of NOP2, especially in ACC and KIRC. Consistent with our results, Wang et al. (2021) demonstrated significant associations between high NOP2 expression and age and tumor stage in KIRC. However, NOP2 expression was not related to age, gender, and tumor stage in resected LUAD, which was not in accordance with our study (Sato et al., 1999). We speculated that this may be caused by very few samples (*n* = 74). These results suggested that NOP2 played an important role in the carcinogenesis and progression in cancers.

It is generally believed that the protein expression level can better reflect the tissue activity of NOP2. Due to the lack of protein expression data in the public database, it is difficult to carry out relevant analysis at the protein level. However, by comparing the transcriptional level with the NOP2 activity, the transcriptional level of most cancers (BLCA, BRCA, CHOL, COAD, ESCA, GBM, HNSC, KIRC, KIRP, LIHC, LUAD, LUSC, PRAD, READ, SARC, STAD, THCA, and UCEC) matches the overall activation of NOP2, indicating that the transcriptional level represents the activation of NOP2 in these cancers. Moreover, the activity and expression of NOP2 were higher in tumor tissues than in normal tissues.

In order to further explore the potential value of NOP2, we explored the correlation between NOP2 expression and the TME. The TME, which contained immune cells such as T and B lymphocytes, natural killer (NK) cells, macrophages, polymorphonuclear cells, dendritic cells, and mast cells, plays a crucial role in tumor progression, invasion, metastasis, immunotherapy response, and immune escape (Dunn et al., 2004; Pitt et al., 2016; Fridman et al., 2017). Our results showed that NOP2 expression correlated negatively with the stromal score in ESCA and LUSC and positively with the immune score in THYM. NOP2 expression also correlated markedly negatively with infiltrating levels of T-cell CD4 memory resting in TGCT and mast cells resting in THYM. In THYM, a positive association was found in T-cell CD4 memory activated. These results demonstrated that NOP2 played a non-negligible role in shaping TME landscapes, implying that NOP2 may affect the therapeutic efficacy of immune checkpoint blockade. Among various immune inhibitors, PVRL2 (CD112) exhibited a significant positive association with NOP2 in UVM. The CD112-CD112R pathway plays a vital role in regulating the process by which T cells kill tumor cells (Murter et al., 2019). In terms of immune stimulators, CD276 (B3H7) exhibited the most significant positive association with NOP2 in UVM. B7-H3 expression in tumor cells contributes to CCL2-CCR2-M2 macrophage axis-mediated immunosuppression and tumor progression (Miyamoto et al., 2022). As for MHC molecules, most of the modulators exhibited a negative correlation with NOP2. Finally, several eligible signaling pathways were identified, containing olfaction, angiogenesis and regulation, RNA polymerase, membrane potential, and transport. All of these identified signaling pathways contributed to further understand the mechanism in pan-cancer.

TMB and MSI played essential roles in cancer tumorigenesis and progression (Hause et al., 2016; Samstein et al., 2019). TMB refers to the number of somatic mutations that occur on the average 1 Mb base in the coding region of the tumor cell genome of tumor cells (Campbell et al., 2017). An elevated mutation burden has been associated with an increased rate of response to anti-CTLA-4 and PD-1 therapies, likely on account of a higher neoantigen burden leading to the antitumor immune response in non-small-cell lung cancer (NSCLC) and UVM (Chan et al., 2015; Rizvi et al., 2015; Chan et al., 2019). MSI refers to the hypermutability of short repetitive sequences in the genome caused by impaired DNA mismatch-repair (MMR) and is a potential predictive marker for immunotherapy (Cortes-Ciriano et al., 2017). MMR deficiency had better response to immune checkpoint blockade and showed improved PFS in COAD and READ (Le et al., 2015). In the current study, NOP2 was positively correlated with TMB and MSI in KICH, LUAD, LUSC, PRAD, and STAD. This indicated that NOP2 might have an indirect effect on the immunotherapeutic response of these cancers. Furthermore, the correlation between NOP2 and the immunotherapeutic response was explored. However, no significant differences were discovered in any of the three cohorts. We speculated that NOP2 may affect the immunotherapeutic response by targeting other immune checkpoints such as CTLA-4, PD-L2, or CD27. As our study only included three immunotherapy cohorts, it could not fully explain the immunotherapeutic response of NOP2. Thus, more immunotherapy-related cohorts should be included in the future.

As far as we know, this is the first study that focuses on the value of NOP2 in pan-cancer. This article systematically shed light on the value of NOP2 in immunotherapy and the relationship between NOP2 and immune modulators, which may help us understand the potential mechanism between NOP2 and the immune system. Of course, our results were based on bioinformatics analysis, meaning that there were no experiments to verify our results, and transcriptomic levels do not reflect protein expression levels or activity. We intend to perform in-depth studies to validate the association between NOP2 and cancer immunotherapy.

## Conclusion

In summary, our outcomes shed light on that NOP2 could serve as a potential clinical and immunotherapeutic predictor in 33 cancers. These findings may provide an immune-based antitumor strategy targeting NOP2.

## Data Availability

The original contributions presented in the study are included in the article/Supplementary Material, further inquiries can be directed to the corresponding authors.

## Abbreviations

m5C: 5-methylcytosine; TCGA: The Cancer Genome Atlas; GEO: Gene Expression Omnibus (GEO); TMB: tumor mutation burden; MSI: microsatellite instability; TME: tumor microenvironment; rRNA: ribosomal RNA; mRNA: messenger RNA; tRNA: transfer RNA; lncRNA: long non-coding RNA; miRNA: microRNA; OS: overall survival; DFS: disease-free survival; DSS: disease-specific survival; PFS: progression-free survival; HR: hazard ratio; ssGSEA: single-sample gene set enrichment analysis; ESTIMATE: Estimation of STromal and Immune cells in MAlignant Tumor tissues using Expression data; CIBERSORT: Cell-type Identification by Estimating Relative Subsets of RNA Transcripts; GSEA: gene set enrichment analysis; CR: complete remission; PR: partial response; SD: stale disease; PD: progressive disease; KM: Kaplan–Meier; GO: Gene Ontology; KEGG: Kyoto Encyclopedia of Genes and Genomes; NK: natural killer; NSCLC: non-small-cell lung cancer; and MMR: mismatch-repair.
