# Supplementary figures and images for: Molecular Characterization Clinical and Immunotherapeutic Characteristics of m5C Regulator NOP2 Across 33 Cancer Types
Source: Front Cell Dev Biol. 2022 Mar 16;10:839136. doi: 10.3389/fcell.2022.839136 (PMC8966037; doi:10.3389/fcell.2022.839136)

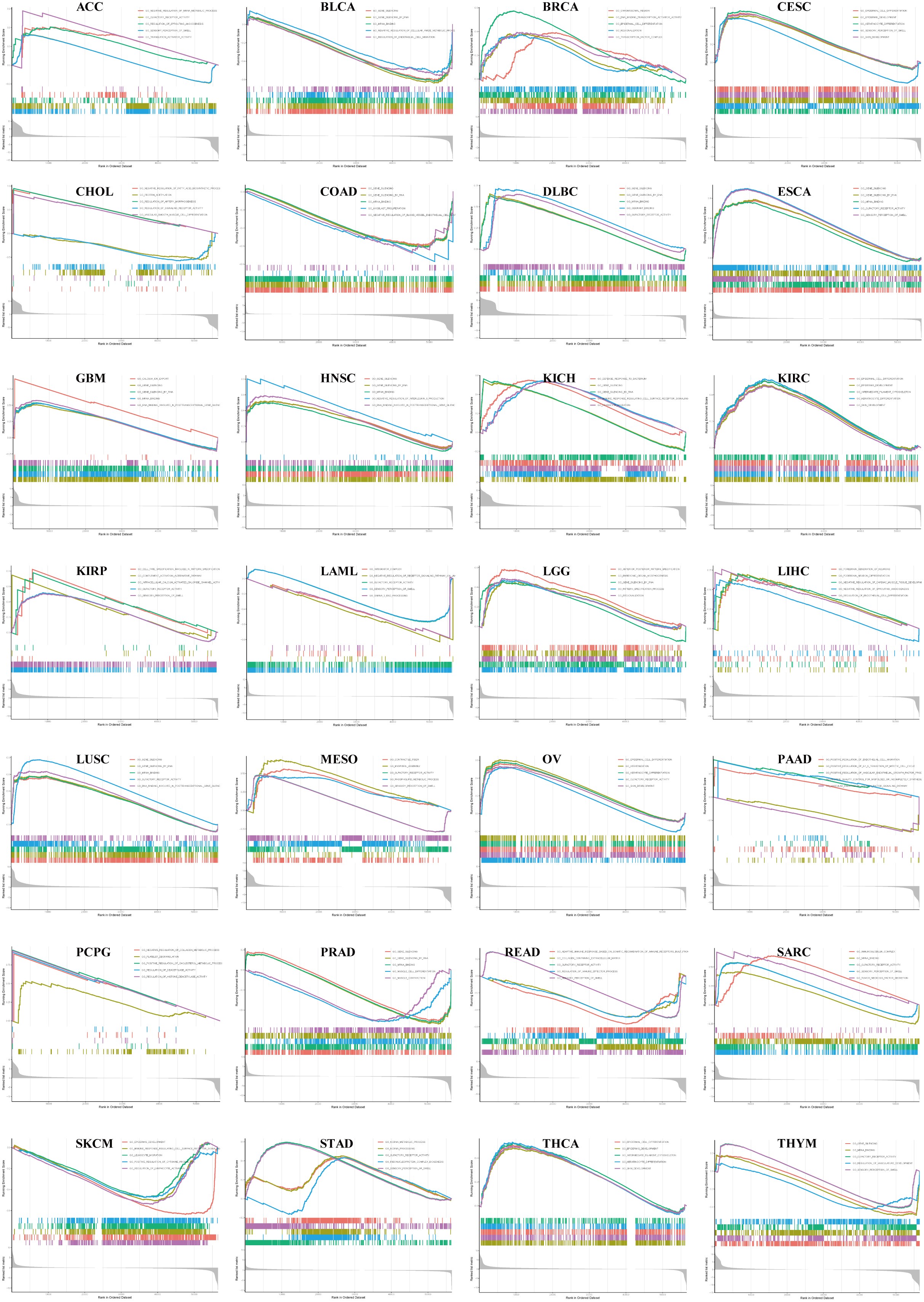

Supplement: Supplementary file 1 [file Image6.TIF]

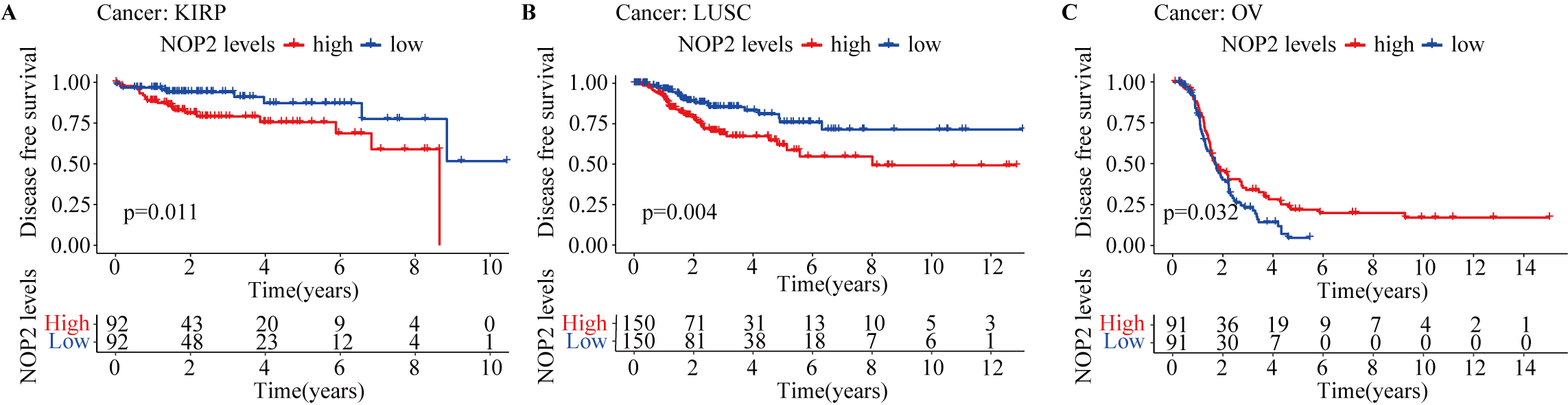

Supplement: Supplementary file 2 [file Image3.TIF]

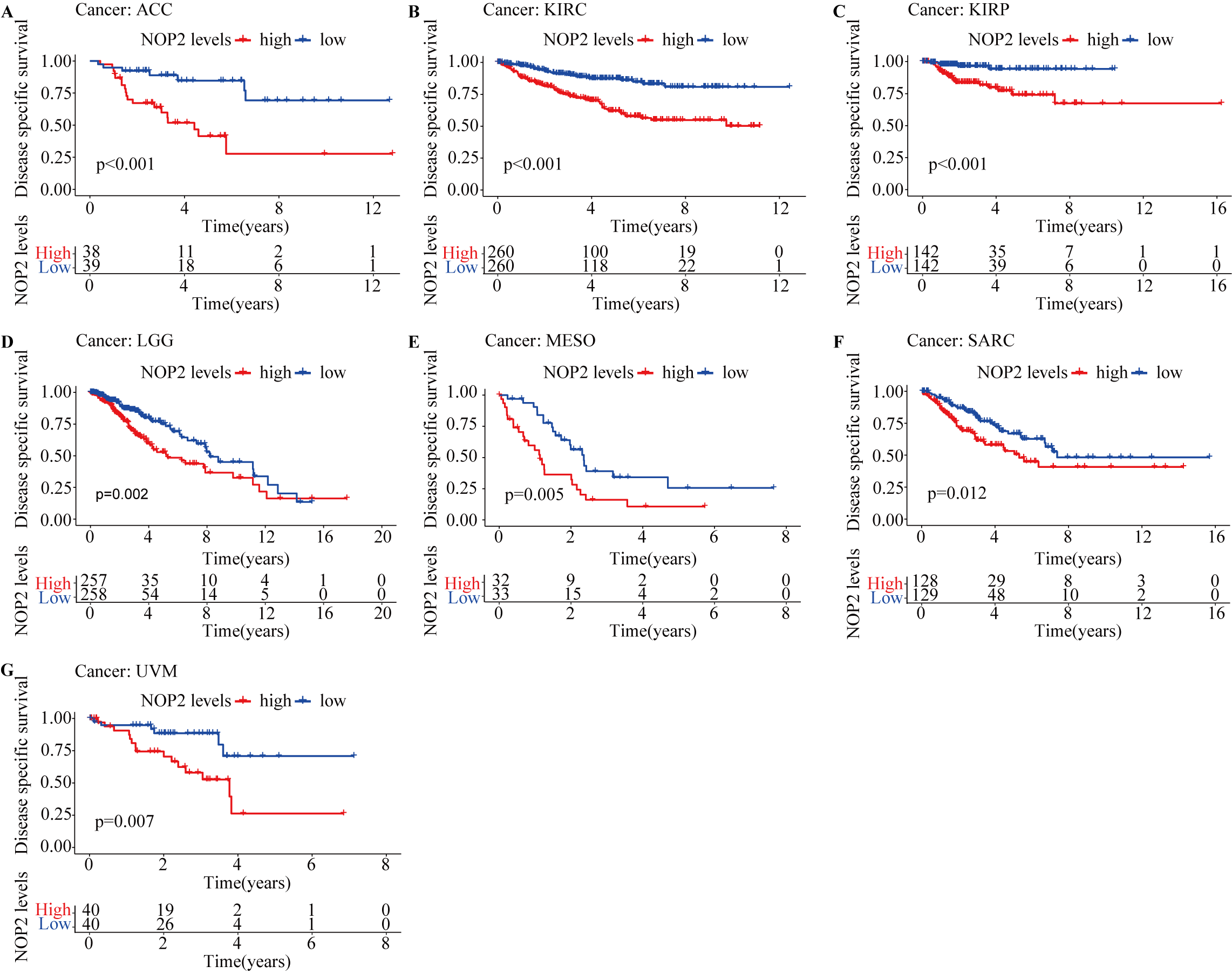

Supplement: Supplementary file 3 [file Image4.TIF]

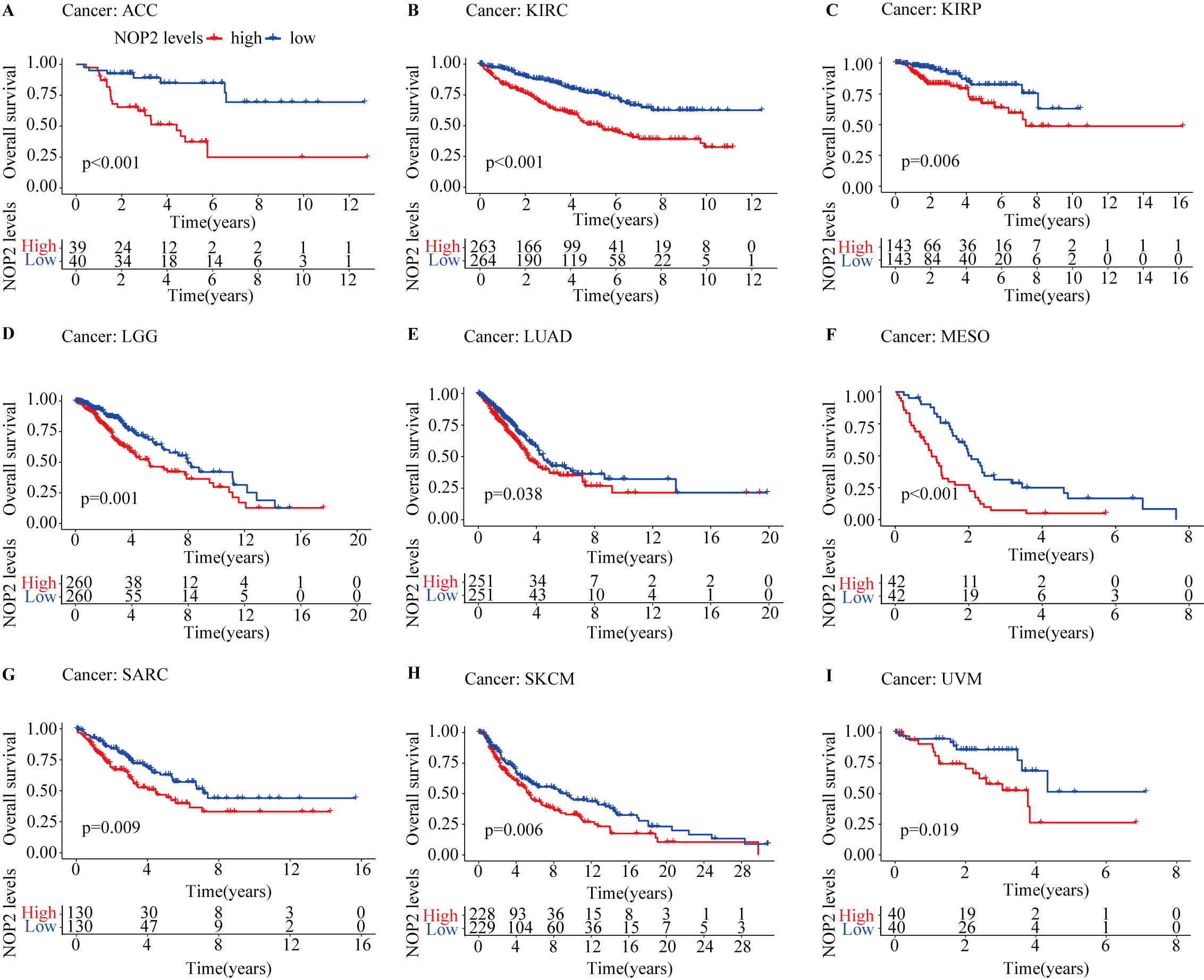

Supplement: Supplementary file 4 [file Image2.TIF]

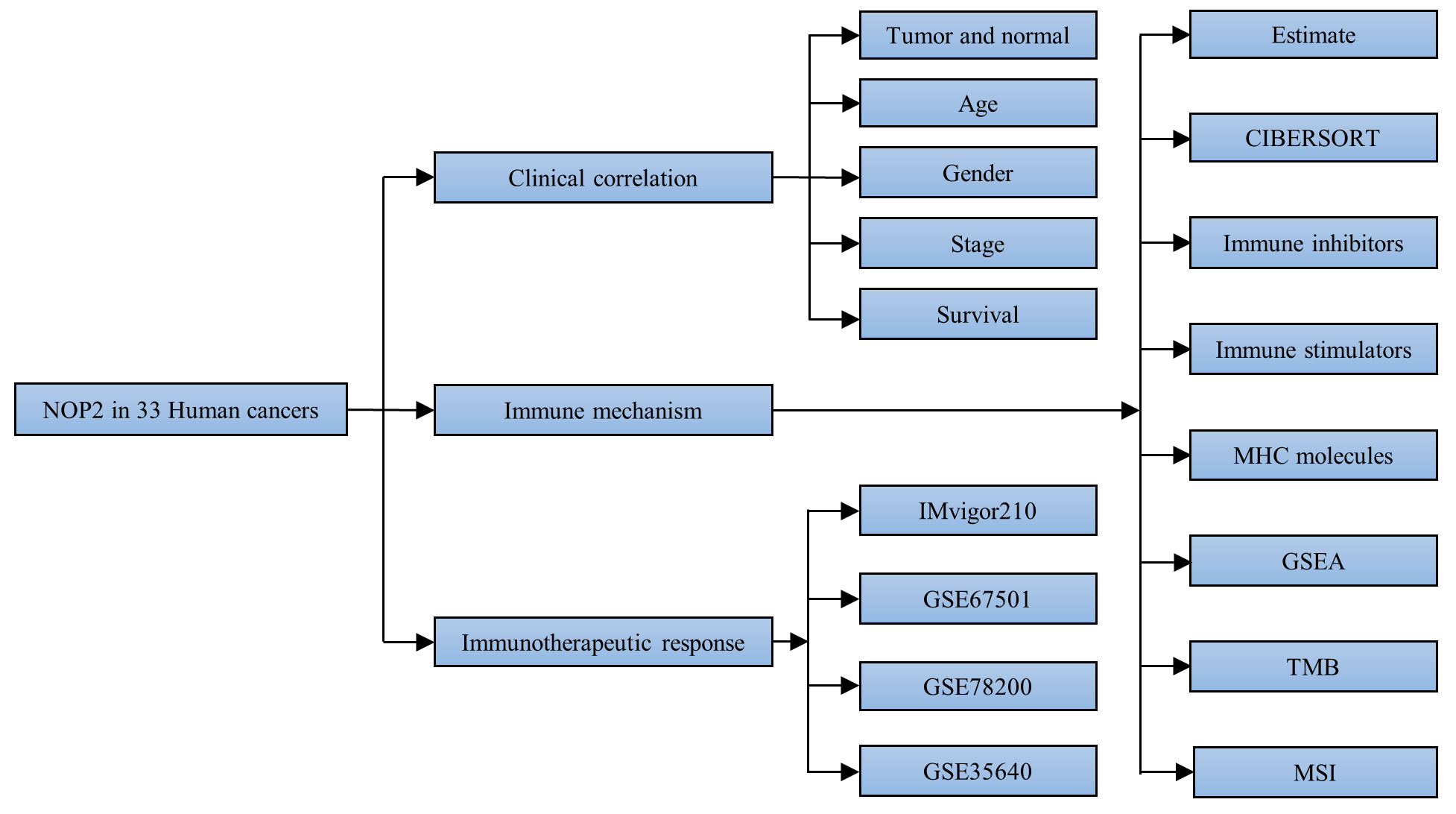

Supplement: Supplementary file 5 [file Image1.TIF]

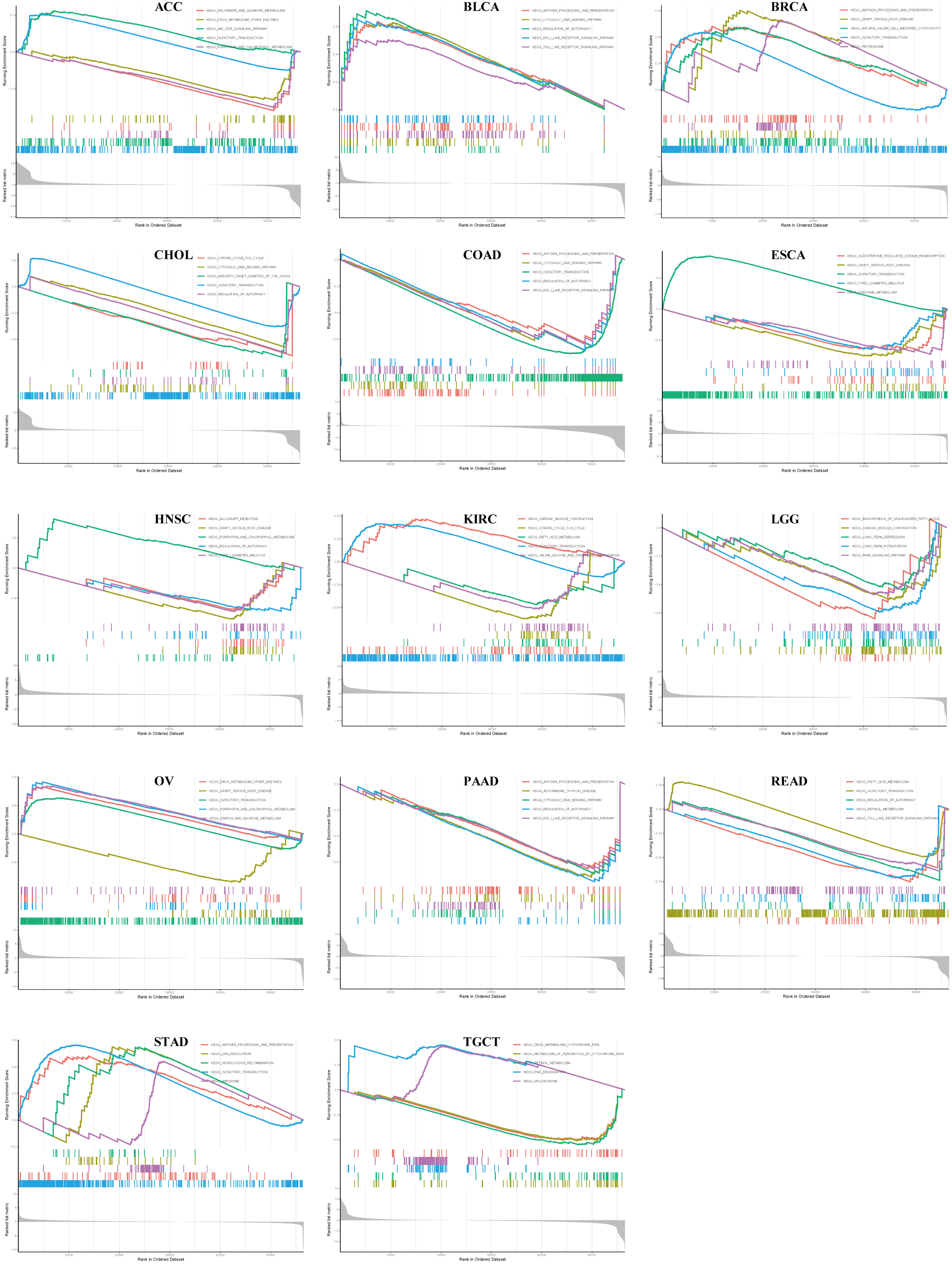

Supplement: Supplementary file 6 [file Image7.TIF]

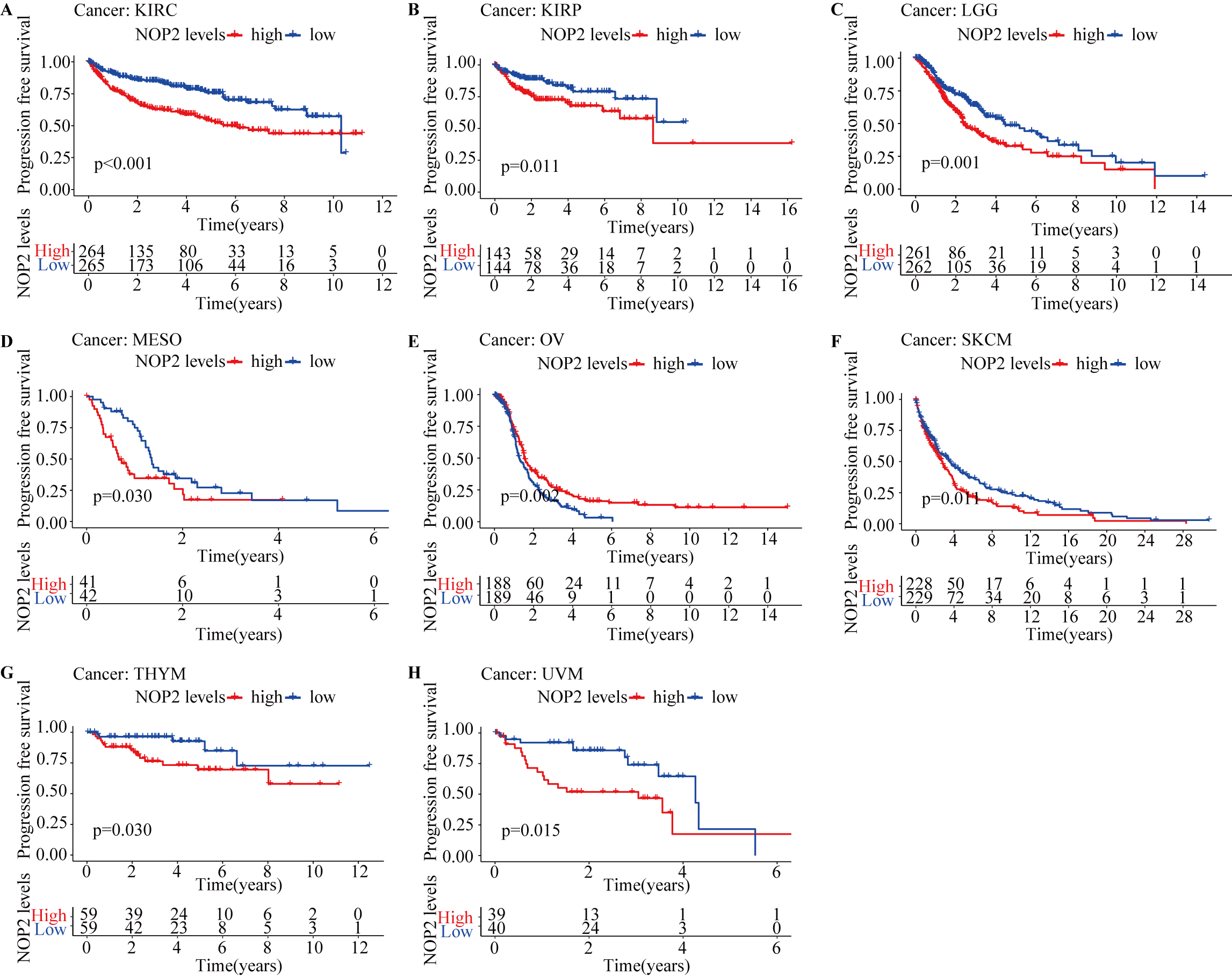

Supplement: Supplementary file 8 [file Image5.TIF]
